# Biosynthesized sulfur nanoparticles: a novel strategy to enhance antioxidant secondary metabolites in *Lotus arabicus* L. callus cultures

**DOI:** 10.1186/s12870-025-06573-z

**Published:** 2025-05-07

**Authors:** Sherien E. Sobhy, Asmaa M. Khalifa, Elsayed E. Hafez, Doaa E. Elsherif

**Affiliations:** 1 Plant Protection and Bimolecular Diagnosis Department, Arid Lands Cultivation Research Institute, City of Scientific Research and Technological Applications, New Borg El-Arab, 21934 Egypt; 2 Botany and Microbiology Department, Faculty of Science, Al-Azhar University (Girls Branch), 11754 , Nasr City, Cairo, Egypt; 3 Botany Department, Faculty of Science, Tanta University, Tanta, 31527 Egypt

**Keywords:** Callus culture, Sulfur nanoparticles, Elicitation, Gene expression, Phenolic, Antioxidant, *Lotus arabicus* L.

## Abstract

**Background:**

Secondary metabolites are distinct compounds with significant medicinal value, yet their production and chemical synthesis present considerable challenges. This necessitates the development of innovative strategies to improve their yield. This study investigated the potential of biosynthesized sulfur nanoparticles (SNPs) as an eco-friendly elicitor to enhance the synthesis of antioxidant secondary metabolites in *Lotus arabicus L.* callus cultures.

**Results:**

After seven weeks, induced calli of *L. arabicus* L were transferred to MS media supplemented with SNPs at different concentrations (0, 25, 50, 100, and 200 mg/l). The results indicated that SNPs (100 mg/l) induced significantly higher profiles for biomass and secondary metabolite compared to the control treatments. Enzyme activities related to secondary metabolite biosynthesis, specifically phenylalanine ammonia lyase (PAL) and polyphenol oxidase (PPO) were enhanced in a dose-dependent manner, with the greatest increases observed at 100 mg/l SNPs. The SNPs also modulated oxidative stress markers (MDA and H_2_O_2_), generally improving callus growth conditions by reducing oxidative stress, except at the highest concentration of 200 mg/l. Additionally, the application of SNPs at 100 mg/l markedly upregulated the expression levels of six crucial genes in the biosynthesis pathway of secondary metabolites (*chalcone synthase* (*CHS), phenylalanine ammonia lyase* (*PAL), flavonol synthase* (*FLS), chalcone isomerase* (*CHI)*, *hydroxycinnamoyl CoA quinate hydroxycinnamoyl transferase (HQT)*, and *deoxyxylulose phosphate reductoisomerase* (*DXR*)). Quantitative HPLC profiling of 16 phenolic and flavonoid compounds revealed that supplementation with SNPs resulted in noticeable boots in the majority of the measured compounds with SNP supplementation.

**Conclusion:**

Overall, the supplementation of SNPs in the culture media of *L. arabicus* L callus positively influenced secondary metabolite production at the molecular and physiological levels, increasing its potential for medicinal use.

## Background

*Lotus arabic L.* is a wild, annual medicinal herb belonging to the lotus genus and family Fabaceae [1]. Folk medicine has traditionally used it for its anti-inflammatory, antimicrobial, and antioxidant qualities, particularly in the treatment of inflammatory and digestive disorder [1, 2]. In addition, *L. arabicus* demonstrating the highest effect against prostate, colon, and breast cancers [1]. Numerous flavonoids of the flavonol type, such as quercetin and kaempferol [3], rutin, and vitexin [4], are known to be present in lotus species, including *Lotus arabicus*. Because *L. arabicus* has a wealth of significant bioactive components, including alkaloids, phenolics, flavonoids, sterols, saponins, tannins, cardiac glycosides, and carbohydrates, it is utilized in traditional and folk medicine [1, 4]. Plant tissue cultures can efficiently produce secondary metabolites with consistent yield and quality [5]. Enhancing the levels of these metabolites can be achieved by optimizing the culture medium's composition and conditions, and adding precursors and elicitors.

Numerous bioactive secondary metabolites that are produced by plants are essential for their survival and environmental adaptation. The three main groups of secondary metabolites found in plants are as follows: alkaloids, terpenoids, and polyphenolics [6]. Polyphenols, the largest class of secondary metabolites identified in plants, are further subdivided into numerous categories with distinct properties, such as flavonoids, phenolic acids, lignans, and stilbenes [7]. These polyphenolic substances are called phenylpropanoids because they are produced from phenylalanine. Flavonoids and phenolic compounds have numerous beneficial biological effects, such as antioxidant [8], anticancer [9], anti-inflammatory [10], and antidiabetic effects [11]. One of the most abundant phenolic acids is chlorogenic acid (CGA) [12]. There are three proposed biosynthetic pathways for the generation of CGA, with the Hydroxycinnamoyl-CoA quinate transferase (HQT)-mediated pathway being reported as the principal method for CGA synthesis [13]. CGA has garnered significant research interest because of its many favourable properties, such as antioxidant, anti-inflammatory, neuroprotective, cardioprotective, and antidiabetic effects [14]. Terpenoids include a wide variety of plant primary and secondary metabolites, including as pigments used in photosynthetic processes, phytohormones, and essential oils [15, 16]. *L. arabicus* L. has been reported to have high amounts of the terpenoid sitosterol, which has many health benefits [1]. Deoxyxylulose phosphate reductoisomerase (DXR), a crucial enzyme in the methylerythritol phosphate (MEP) pathway, catalyzes the transformation of deoxyxylulose phosphate (DXP) into MEP, the shared substrate for terpenoid biosynthesis [17]. It has been demonstrated that activating DXR improves the terpenoid pathway, and it is thought to be a crucial regulatory enzyme in regulating the manufacture of terpenoid [16, 18].

Researchers can guarantee a consistent and sustainable supply of bioactive compounds by utilizing cutting-edge technological approaches like tissue culture and nanoparticle-based techniques [19, 20]. This will lessen reliance on wild collection and increase the possibility of using natural herbal remedies for many illnesses. It has been established that in vitro plant cell and callus culture methods are beneficial for generating increased amounts of secondary metabolites [20]. Tissue culture methods for plants including callus and in vitro propagation ensure the production rate of secondary compounds in higher quantities without inhibiting the effects of the atmosphere [5, 21]. By optimizing the culture medium's composition, adding precursors and elicitors, and setting up the right growth conditions, the amount of secondary chemicals in cell and organ cultures was greatly increased [22].

Elicitation is a popular and economical method by which plants produce secondary metabolites by inducing chemical defense behavior. It uses a variety of physiological and chemical elements called elicitors [23]. The use of elicitation can maximize productivity in small quantities of cultures while shortening the time needed for secondary metabolite synthesis [24]. However, elicitation effectiveness is contingent upon the type, dose amount, elicitor specificity, and cultural context [25, 26]. Nanoparticles may function as elicitors and sources of nutrients when they are introduced in to the in vitro growth media of plant. Researchers have demonstrated that NPs can improve the secondary metabolism of plant callus cultures [27]. Sulfur is a critical micronutrient element that is required for the production of several biomolecules, such as amino acids, coenzymes, vitamins, proteins, and chlorophylls [28] Sulfur also helps plants become resistant to abiotic stressors and illnesses [29]. The applications for sulfur in industry, agriculture, and medicine are numerous and include the creation of fungicides, antimicrobial agents, and fertilizers [30, 31]. The present study is likely among the first to investigate the potential of SNPs in plant tissue culture applications. Therefore, the present work investigates a novel method to address the ongoing challenge of producing considerable quantities of valuable medical bioactive compounds. By utilizing biosynthesized SNPs as elicitors, we sought to develop a sustainable and efficient strategy for increasing the synthesis and production of the secondary metabolites in *L. arabicus* L. calli.

## Materials and methods

### Plant material and callus induction

The origin of the *Lotus arabicus* L seeds was the Al-Azhar gardens in Egypt (30.0408°N, 31.2647°E), during the fruiting stage between May to July 2023. They were identified by Dr. Iman H. Al-Gohary from Herbarium Unit, Desert Research Centre (DRC), and Cairo, Egypt. Herbarium specimens were deposited in Tanta University Herbarium (TANE) with accession number 14306. The seeds were surface sterilized in laminar air flow hood by dipping them in 70% ethanol for one minute and then immersing them in a 20% Clorox solution containing 5.4% sodium hypochlorite (NaOCl) and drops of any detergent for 15 min. Finally the samples were rinsed five times with sterilized distilled water.

**Fig. 1:**
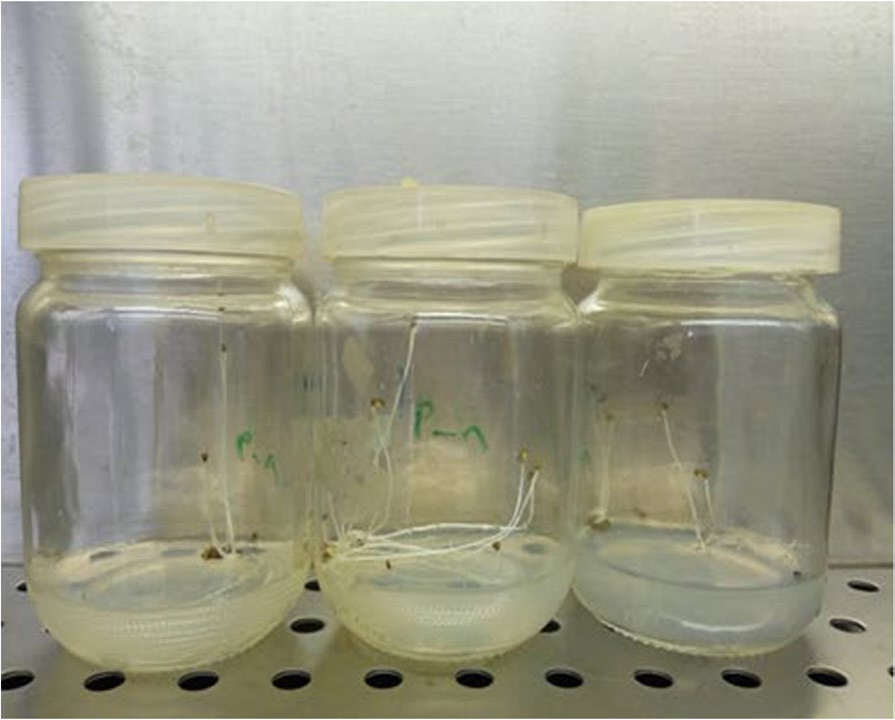
Germinated seeds of* L. arabicus* on half strength MS media

The sterilized seeds were inoculated on half-strength Murashige and Skoog, [32] media fortified with 30 g/l sucrose and 8 g/l agar (Fig. 1). To aid in germination, the jars were incubated at 25 ± 2 °C in the dark. For callus induction, 1 cm segments of stem seedling (10 day old) from germinated seeds were cultured on solid MS media supplemented with 1 mg/l 2,4-D (2,4-dichlorophenoxy acetic acid) and 1 mg/l BA (6-benzyladenine).

### Culture medium and conditions

After seven weeks, the successfully generated callus were transferred to MS media (1 mg/l, 2,4-D, and 1 mg/l BA) fortified with different concentrations of SNPs (0, 25, 50, 100, and 200 mg/l), and callus were then incubated in the dark at 25 °C with ten replica for each treatments.

### Sulfur nanoparticles synthesis and characterization

The characterization of the green synthesized SNPs nanoparticles, via XRD (GNR-APD 2000 pro), an FT-IR spectrophotometer (Bruker Tenor 27), UV–Vis spectrophotometer (Shimadzu 240), and a transmission electron microscope (JEOL, JEM- 2100, Tokyo, Japan) was reported previously Khalifa et al., [33]. Therefore, the average size of the synthesized SNPs was 10.98 ± 2.91 nm.

### Physiological assessments

#### Biomass formation

The calli were collected after seven weeks, and the fresh weights were measured. The fresh samples were then dried for 48 h at 40 °C, after which their dry weight was determined.

### Antioxidant compounds (phenolic, flavonoids and ascorbic)

#### Estimation of phenolic contents

A quantitative estimation of the total phenolic content was made via Jindal and Singh [34] approach. The extract was prepared with 100% ethanol; 1 ml of this extract was then combined with 1 ml of Folin-Ciocalteu,s reagent and 1 ml of Na_2_CO_3_ (20%), then the mixture was further reconstituted to a known volume with distilled water. After 30 min, the absorbance of the mixture was measured at 650 nm against a gallic acid standard curve (0–1 mg/l, *R*^2^ = 0.9942).

#### Estimation of flavonoids

Flavonoids were measured via a colorimetric approach with aluminum chloride [35]. An aliquot of ethanolic callus extract was combined with 10% aluminum chloride, 1 M potassium acetate, and 95% ethanol. The mixture was then brought to an appropriate volume with distilled water and allowed to sit at room temperature for 30 min. The absorbance was determined at 417 nm and compared to a standard of quercetin curve (0–1 mg/l, *R*^2^ = 0.9848).

#### c- Determination of ascorbic acid (AsA)

The non-enzymatic antioxidant ascorbic acid was calculated via Oser [36]. Callus tissues were ground with 5% sulfosalicylic acid. The reaction mixture was composed of 1.5 mM Na_2_HPO_4_, 0.15 N H_2_SO_4_, 2% Na-molybdante, and callus extract. After 40 min of incubation at 60 °C in a water bath, the mixture was cooled and centrifuged, then absorbance was determined at 660 nm as mg/g FM.

#### Estimation of enzymes

Fresh *L. arabicus* tissue calli were homogenized with liquid nitrogen, extracted with cold phosphate buffer (pH 7) and centrifuged at 5000 rpm. Two important enzymes, polyphenol oxidase (PPO) and phenylalanine ammonia lyase (PAL) [EC1.10.3.1] were assayed in the supernatant.

The assay for phenylalanine ammonia lyase (PAL) was conducted via El-Shora [37] the protocol. About 0.45 ml of 100 mM Tris–HCl buffer (pH 8.5) with 1 mM β-mercaptoethanol, 0.5 ml of 50 mM L-phenylalanine, and 0.5 ml of enzyme extract were added to 1 mL of the reaction mixture and then incubated for 15 min at 30 °C. To stop the reaction, 100 μL of 6 M HCl was added. Using the reaction mixture devoid of the enzyme extract as a blank, the absorbance was determined at 290 nm using spectrophotometry.

PPO activity was measured using the extinction coefficient of 26.40 M- 1 cm- 1 and purpurogallin production was monitored at 420 nm, as per the protocol of Kumar and Khan [38]. Adding the enzyme extract was added to the reaction mixture (2 mM pyrogallol in 0.1 M K-phosphate buffer, pH 6), the mixture was allowed to react for 5 min at 25 °C. The reaction was then stopped with H_2_SO_4_ (2.5 N), and the absorbance was measured at 420 nm. The activity of each enzyme was reported in units of µM/g FM min^−1^.

#### Evaluation of stress biomarkers

H_2_O_2_ and malondialdehyde (MDA) levels were measured in accordance with the methods of Velikova et al. [39]and Heath and Packer [40], respectively. Fresh callus tissue (0.1 g) was ground in 0.1% trichloroacetic acid (TCA) to determine H_2_O_2_. After the callus extract was mixed with KI_2_ (1M) and phosphate buffer (pH 7.0), the absorbance was measured at 390 nm. callus samples were ground in a 5% (w/v) TCA solution to quantify MDA, a byproduct of lipid peroxidation. The sample extract and 0.67% (w/v) TBA made up the reaction mixture, which was heated for 20 min before being chilled. The absorbance was measured at two wavelengths: 532 nm and 600 nm.

#### HPLC conditions

The *L. arabicus L* ethanol extract was prepared for HPLC analysis via an Agilent 1260 series. The separation was carried out via an Eclipse C18 column (4.6 mm × 250 mm i.d., 5 μm) for flavonoid and phenolic compound analysis. The separation of flavonoid and phenolic acids was carried out with a mobile phase consisting of 0.05% trifluoroacetic acid in acetonitrile (A) and water (B) at a flow rate of 0.9 ml/min. The mobile phase was programmed consecutively in a linear gradient as follows: 0 min (82% A); 0–5 min (80% A); 5–8 min (60% A); 8–12 min (60% A); 12–15 min (82% A); 15–16 min (82% A) and 16–20 (82%A). The multi-wavelength detector was monitored at 280 nm. The injection volume was 5 μl for each of the sample solutions. The column temperature was maintained at 40 °C.

### Molecular estimation

#### Total RNA extraction, cdna synthesis and RT-PCR

Total RNA was isolated from *L. arabicus* calli via the RNeasy Mini Kit (Qiagen). Through reverse RNA transcription, 20 μl of complementary DNA (cDNA) was obtained via a thermocycler (MJ Research, Inc., PTC- 100 TM Programmable thermal controller, USA). The first enzyme activation cycle at 42 °C for one hour and the second enzyme inactivation cycle at 95 °C for five minutes were part of the reaction conditions.

SYBR Green PCR Master Mix (Fermentas, USA) was used to perform qRT-PCR in triplicate. For each reaction, a 25 μl mixture including the primer pairs (*CHI, CHS, PAL, FLS, HQT,* and *DXR*) listed in Table 1 was utilized. Data were collected during the extension stage. Using a Rotor-Gene 6000 (QIAGEN, ABI System, USA), the reaction was carried out. the The relative expression of the studied genes was quantified and calculated made according to Livak and Schmittgen; Sobhy et al. [41, 42].

**Table 1:** Specific primer sequences used in this study

**Gene**	**Primer sequences 5′ــــــــــــــــــــــــ 3′**
*Reference gene (ß-Actin)*	F: 5'GTGGGCCGCTCTAGGCACCAA3'
	R:5'CTCTTTGATGTCACGCACGATTTC3'
*Phenylalanine ammonia lyase (PAL)*	F: 5′ GCAAGGAAAGCCCGAGTTTAC3'
	R: 5′ GGACCTTTTTGGCTACTTGGC3'
*Chalcone synthase (CHS)*	F: 5'-AAGCTCTCACTCTCCGGT3'
	R: 5'- TCGTGTGAGTCCCTTGCT3'
*Chalcone isomerase (CHI)*	F: 5'TGGTGGCCTAGACAACGATGAGTT3'
	R: 5'TCACACTCCCAACTTGGTTTCCCT3'
*Flavonol synthase (FLS)*	F: 5'TTAAAGGAAGGTCTCGGTGGCGAA3'
	R: 5'TCATTGGTGACGATGAGTGCGAGT3'
*hydroxycinnamoyl CoA quinate*	F: 5'CCCAATGGCTGGAAGATTAGCTA3'
*hydroxycinnamoyl transferase (HQT)*	R: 5'CATGAATCACTTTCAGCCTCAACAA3'
Deoxy-D-ylulose 5-phosphate reductoisomerase (DXR)	F: 5'CTTGTTGAAAAGTGTGGCAGAG3'
	R: 5'TTGGTTGCTGAGCTAAAAGAAG3'

### Statistical analysis

The findings are presented as the standard deviation (SD) plus the mean of three replicates. XLSTAT software (version 2014.5.03) revealed significant differences (p < 0.05) between the treatments for the various measured variables when one-way variance (ANOVA) was used to evaluate the differences. Duncan's test was then used to confirm the findings. XLSTAT software was used to calculate the Pearson correlation coefficient and conduct principle component analysis (PCA) between various metrics and SNP treatments.

## Results

### Changes in the biomass of SNPs-treated and untreated *Lotus arabicus* L calli

**Fig. 2:**
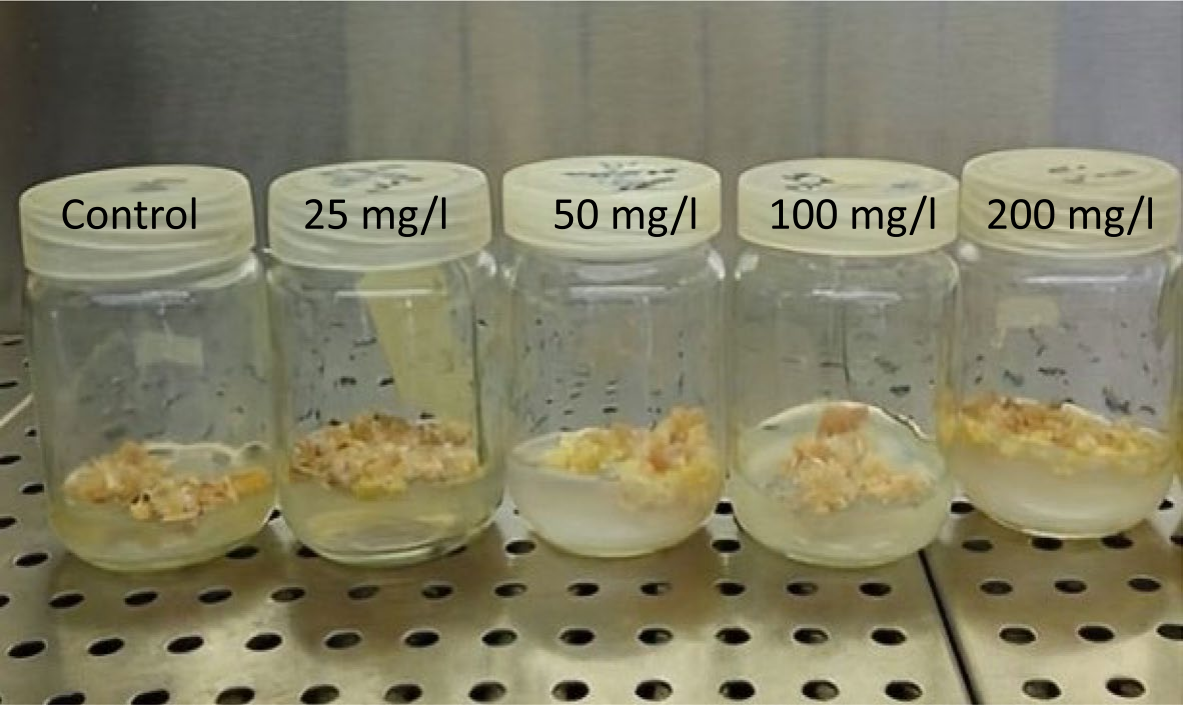
Effect of different SNPs conc. (25, 50, 100 and 200 mg/l) on *L. arabicus* L

**Fig. 3:**
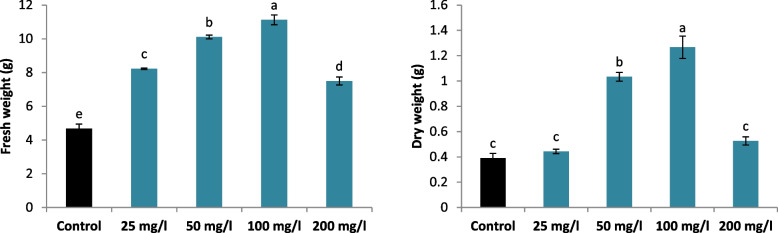
Effect of different SNPs conc. (25, 50, 100 and 200 mg/l) on the biomass (fresh weight and dry weight) of *L. arabicus* L

The biomass development of *L. arabicus* L calli is greatly impacted by the addition of biosynthetic SNPs to the growth medium in an optimally administered dosage manner (Fig. 2). The callus fresh weight and dry weight gradually rose after the SNPs were added to the media. Furthermore, in comparison with the control, 100 mg/l supplementation resulted in the highest callus fresh weight and dry weight (137.7 and 224.4%, respectively) (Fig. 3).

### Changes in the levels of flavonoids, phenolic, and ascorbic acid in SNPs-treated and untreated L. arabicus L callus

**Fig. 4:**
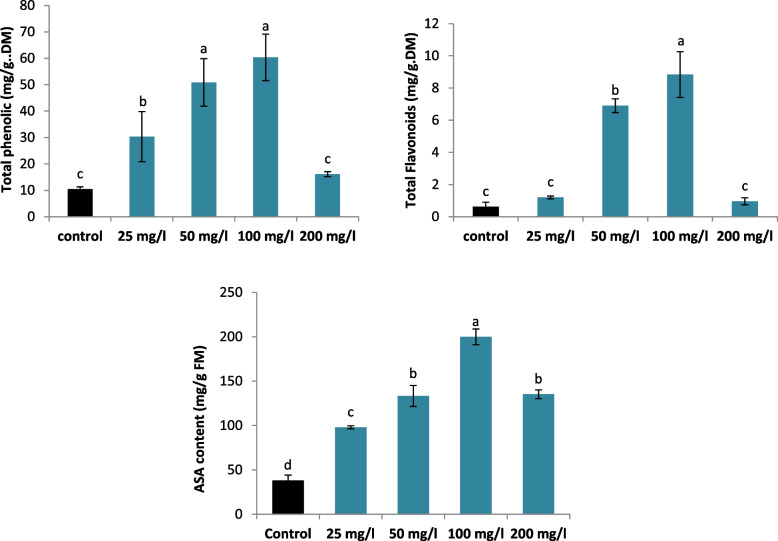
Effect of different SNPs conc. (25, 50, 100 and 200 mg/l) on the phenolic, flavonoid and ascorbic acid contents of *L. arabicus* L

Compared with no supplementation, the addition of SNPs to *L. arabicus *media significantly elevated the phenolic level. A maximum increase of 478.78% in phenolic content was recorded in 100 mg/l SNPs-treated plants. In comparison to the control, callus treated with 200 mg/l SNPs exhibited a non-significant rise in the phenolic content of 54.54%. Compared with the control, only both 50 and 100 mg/l SNPs treatments significantly increased of the flavonoid content by 1021.42% and 1334.28% in flavonoid content, respectively. Furthermore, the presence of SNPs greatly raised the ascorbic acid level in *L. arabicus* L media and this increase was greater by 423.1% in the 100 mg/l treatment than in the control treatment (Fig. 4).

### Enzymes activities

**Fig. 5:**
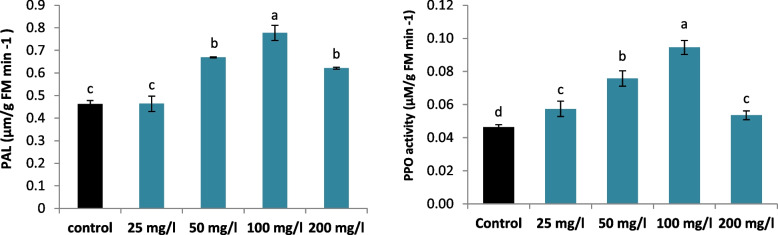
Effect of different SNPs conc. (25, 50, 100 and 200 mg/l) on PAL and PPO activities of *L. arabicus* L

Compared with the control, SNPs supplementation significantly improved PAL and PPO activities in comparison in a dose-dependent manner. Compared with those control treatment, the increase in PAL activity was 12.21%, 44.67%, 68.14% and 43.35%, and the increase in PPO activity was 23.96%, 63.56%, 104.14% and 15.66%, under 25, 50, 100 and 200 mg/l SNPs treatments, respectively. Maximum increases of 68.14% in PAL and 104.14% in PPO were recorded in calli grown with 100 mg/l SNPs. The activities of PAL and PPO declined after reaching a maximum in the 200 mg/l SNPs treatment (Fig. 5).

### Changes in the membrane stability marker (MDA) and signaling molecule (H2O2) contents of SNPs-treated and untreated *L. arabicus* L calli

**Fig. 6:**
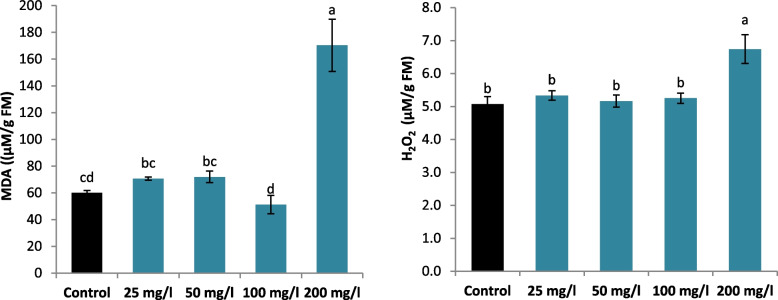
Effect of different SNPs conc. (25, 50, 100 and 200 mg/l) on MDA and H_2_O_2_ contents of *L. arabicus* L

Compared with control, SNPs supplementation did not considerably alter the MDA content in treated *L. arabicus* L calli, except at 200 mg/l SNPs, which significantly increased the MDA content by 184.13% (Fig. 4). In addition to application of SNPs at 200 mg/l markedly increased H_2_O_2_ accumulation with a maximum increase of 32.84% in H_2_O_2_ content, compared to control (Fig. 6).

### Changes in the genes expression levels in SNPs-treated and untreated L. arabicus L callus

**Fig. 7:**
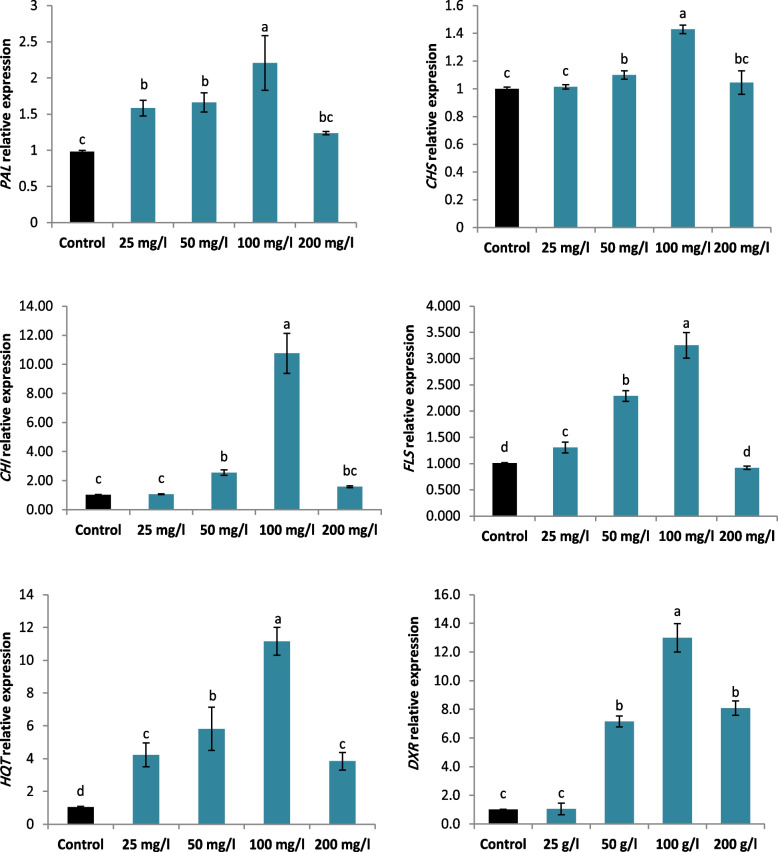
Effect of different SNPs conc. (25, 50, 100 and 200 mg/l) on *PAL*, *CHS*, *CHI*, *FLS*, *HQT* and *DXR* relative expression in *L. arabicus* L callus

To prolong our investigations of the physiological responses of *L. arabicus* L callus to different doses of SNPs, fold changes in the expression levels of certain genes involved in the plant flavonoid biosynthetic pathway were observed (Fig. 7). The findings demonstrated that, in comparison with those in the control, the expression levels of the genes *PAL, CHS, CHI*, and *FLS* were significantly higher in the calli supplemented with SNPs. The highest change in the expression of these genes was recorded in 100 mg/l SNP-supplemented with values of 1.2, 0.4, 9.73 and 2.24 fold, respectively. In addition, the expression levels of gene involved in the chlorogenic acid biosynthetic pathway (HQT) significantly changed especially those encoding in 100 mg/l SNPs (10.11 fold). Similarly, calli treated with 100 mg/l SNPs showed the highest expression levels of *DXR* gene of terpenoid biosynthetic pathway (11.9 fold).

### Quantitative analyses of phenolic acids and flavonoids

HPLC analysis allowed the identification of sixteen constituents, namely; ten phenolic compounds (gallic acid, methyl gallate, coffeic acid, syringic acid, ellagic acid, coumaric acid, vanillin, ferulic acid, daidzein, and cinnamic acid), and six flavonoids (chlorogenic acid, catechin, rutin, naringenin, quercetin, and apigenin) in SNPs-treated and untreated *L. arabicus* L calli (Table 2). The results of HPLC quantitative profiling revealed that phenolic and flavonoid compounds were increased in SNPs treatments especially at 100 mg/l (1.1fold), compared with those in the control. Additionally, the present data revealed highly increased amounts of benzoic acid derivatives, such as gallic acid (1.37fold), methyl gallate (22.9fold), and syringic acid (2.4fold) under 100 mg/l SNPs treatment, compared with the control, whereas the highest ellagic acid and vanillin concentrations were recorded at 25 mg/l and 50 mg/l SNPs, respectively. Moreover, compared with those in the control, concentrations of cinnamic acid derivatives, such as coumaric acid (fivefold), coffeic acid (0.92 fold) and chlorogenic acid (1.17fold), were increased in SNPs treatments at 100 mg/l.

**Table 2:** Chemical composition of control and SNPs (25, 50, 100 and 200 mg/L) treated *L. arabicus* L subjected to HPLC

**No**	**Category**	**Compounds**	**Control**	**25 mg/l**	**50 mg/l**	**100 mg/l**	**200 mg/l**	**RT**
			**µg/ml**					**min**
1	**Phenolics**	Gallic acid	4.15 ± 0.2^d^	6.26 ± 0.1^b^	5.93 ± 0.5^c^	9.86 ± 0.6^a^	6.55 ± 0.3^b^	3.38
2		Methyl gallate	0.11 ± 0.3^d^	0.29 ± 0.1^d^	1.46 ± 0.5^b^	2.63 ± 0.4^a^	0.45 ± 0.1^c^	5.54
3		Coffeic acid	1.38 ± 0.8^c^	1.4 ± 0.3^c^	1.9 ± 0.7^b^	2.65 ± 0.5^a^	1.6 ± 0.5^b^	6.09
4		Syringic acid	0.27 ± 0.01^c^	0.29 ± 0.7^b^	0.34 ± 0.8^ab^	0.93 ± 0.1^a^	0.29 ± 0.2^c^	6.55
5		Ellagic acid	1.96 ± 0.3^c^	8.28 ± 1.1^a^	4.16 ± 0.9^b^	0.5 ± 0.1^d^	4.0 ± 0.9^b^	8.54
6		Coumaric acid	0.07 ± 0.01^c^	0.09 ± 0.02^c^	0.16 ± 0.03^b^	0.42 ± 0.1^a^	0.14 ± 0.04^b^	9.21
7		Vanillin	0.23 ± 0.05^b^	0.15 ± 0.01^c^	0.41 ± 0.2^a^	0.32 ± 0.03^b^	0.35 ± 0.09^b^	9.77
8		Ferulic acid	0.07 ± 0.01^d^	0.44 ± 0.3^a^	0.22 ± 0.1^b^	0.12 ± 0.05^c^	0.11 ± 0.1^c^	10.05
9		Chlorogenic acid	6.14 ± 0.34^c^	6.17 ± 0.5^c^	8.6 ± 1.2^b^	13.88 ± 1.6^a^	5.6 ± 0.8^cd^	4.15
10		Cinnamic acid	0.12 ± 0.01^c^	0.16 ± 0.1^bc^	0.2 ± 0.4^b^	0.3 ± 0.07^a^	0.23 ± 0.6^ab^	14.07
11	**Flavonoids**	Daidzein	0.13 ± 0.03^cd^	ND	0.45 ± 0.5^ab^	0.64 ± 0.4^a^	0.18 ± 0.1^c^	12.16
12		Catechin	0.67 ± 0.1^c^	0.72 ± 0.6^bc^	0.95 ± 0.7^b^	1.8 ± 0.4^a^	ND	4.62
13		Rutin	0.62 ± 0.2^c^	ND	0.64 ± 0.1^ab^	0.75 ± 0.4^a^	0.63 ± 0.2^bc^	8.09
14		Naringenin	0.13 ± 0.05^b^	0.13 ± 0.6^b^	0.16 ± 0.4^ab^	0.29 ± 0.8^a^	0.11 ± 0.1^c^	10.45
15		Quercetin	0.57 ± 0.05^cd^	1.42 ± 0.9^ab^	0.99 ± 0.5^bc^	0.84 ± 0.1^bc^	3.13 ± 0.9^a^	12.61
16		Apigenin	0.84 ± 0.6^ab^	ND	0.26 ± 0.1^b^	1.3 ± 0.9^a^	0.2 ± 0.3^c^	14.62
**Total content**			17.87 ± 1.1^e^	26.36 ± 0.9^c^	27.09 ± 1.1^b^	37.28 ± 2.4^a^	24.59 ± 0.4^d^	

*ND* not detected, *RT retention time (minutes)

Different lowercase letters are used to indicate statistically significant differences at Duncan’s test (*P*< 0.05)

Among the flavonoid compounds, 100 mg/l SNPs application increased the major flavonoid compounds, such as daidzein, catechin, rutin, naringenin, and apigenin, by 3.9, 1.17, 0.21, 0.47, and 0.54fold respectively, greater than the control. The concentration of quercetin was showed peaked under 200 mg/l SNPs treatment and was 3.03fold greater than that of the control.

### Correlation profiles

**Fig. 8:**
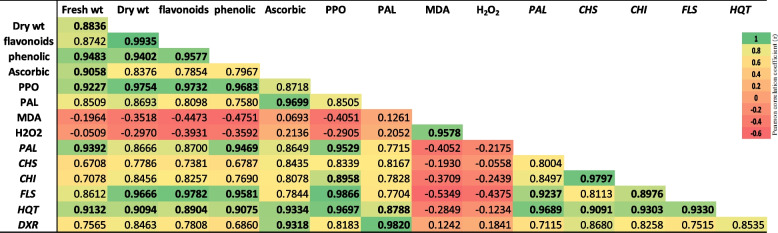
Pearson correlation coefficient for secondary metabolite pathways determined at different concentrations of SNPs treatment in *L. arabicus* L. calli (0, 25, 50, 100, and 200 mg/l)

Pearson's simple correlation was used to analyse the connections between physiological and molecular characteristics observed in various treatments with SNPs (as shown in Fig. 8). The findings revealed strong positive correlations between biomass yield (fresh and dry weight) and phenolic, flavonoid, and ascorbic acid. The phenolic and flavonoid contents were also found to be positively related with augmented expression levels of *PAL, CHS, CHI, FLS* and *HQT* genes. Furthermore, there were positive correlations between the PPO content, phenolic, flavonoids, and genes expression levels, including *CHI, PAL, HQT* and *FLS*. Conversely, there were negative correlations between H_2_O_2_, MDA, and antioxidant activities and secondary metabolites.

### Principal Component Analysis (PCA)

**Fig. 9:**
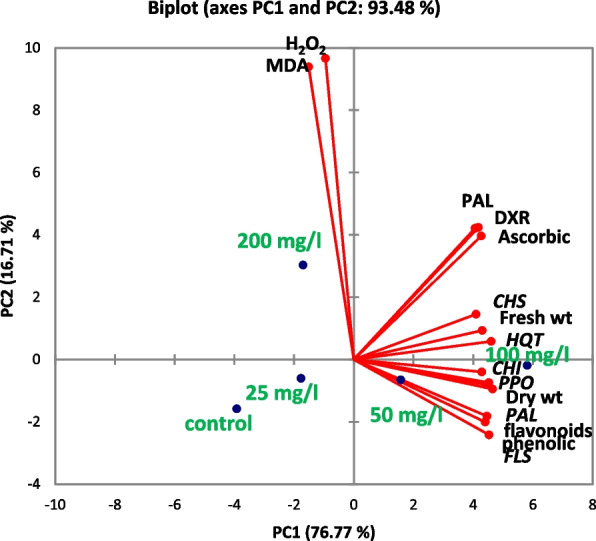
Principal component analysis of the relative expression of genes and physiological characteristics for the route of secondary metabolites assessed at SNPs doses of *L. arabicus* L callus (0, 25, 50, 100, and 200 mg/l)

The results of the PCA linked the treatments of SNPs with physiological factors and the relative expression of genes (Fig. 9). In terms of the overall variation, the first four principle components (PCs) explained 100% of the variation. With eigenvalues > 1, the first four PCs explained 93.43% of the variation. PC1 presented 76.77% of overall variation, which was significantly correlated with the following variables in descending order of strength: PPO, *HQT*, *FLS*, dry weight, flavonoids, *PAL,* phenolics, fresh weight, *CHI,* ascorbic acid, PAL, *CHS*, and *DXR*. In addition, the PCA plot revealed that treatment with 100 mg/l SNPs exhibited high levels of all aforementioned parameters. Furthermore, the second PC explained 16.71% of the overall variation and was the main factor influenced by H_2_O_2_ and MDA. Moreover, the PCA plot revealed that treatment with 200 mg/l SNPs exhibited high levels of these two stress markers.

**Fig. 10:**
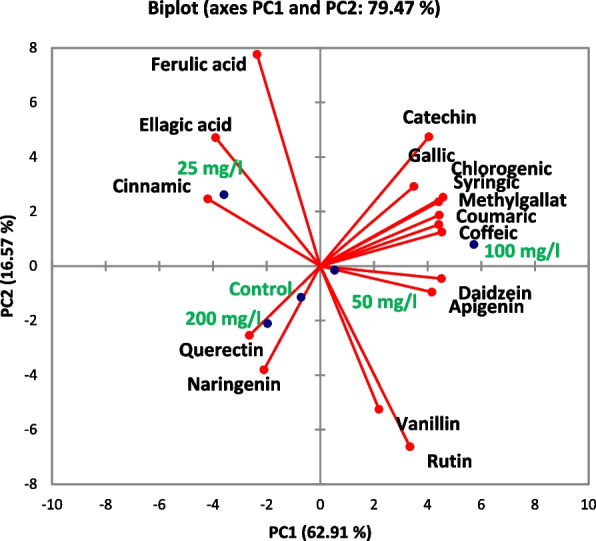
Principal component analysis of flavonoid and phenolic compounds data from SNPs doses of *L. arabicus* L callus (0, 25, 50, 100, and 200 mg/l)

PCA revealed the relationships of phenolic and flavonoid compounds in untreated and treated calli with different SNPs (Fig. 10). The analysis revealed that 79.47% of the variation was explained by the first four PCs with eigenvalues ≥ 1. PC1 was the most significant, presenting 62.91% of the overall variation. The compounds that showed a strong correlation in the PC1 were chlorogenic acid, caffeic acid, daidzein, methyl gallate, coumaric acid, syringic acid, cinnamic acid, apigenin, catechin, ellagic acid, and gallic acid, in descending order. PC2, which noted 16.57% of the overall variation, was most impacted by rutin and ferulic acid. PC3, which explained 14.84%, demonstrated a substantial correlation with both naringenin and quercetin, whereas PC4, which represented 5.68%, was mostly correlated with vanillin.

## Discussion

Nanoparticle-supplemented in vitro plant cultures show promise as a significant technological advancement for the efficient production of valuable secondary metabolites on a large scale [43]. In addition to being secondary metabolite elicitors, nanoparticles can also function as sources of micronutrients, stimulators of callus induction, shoot growth and antimicrobial agents [44]. In accordance with our findings, calli were grown on media supplemented with SNPs at a concentration of 100 mg/l, which resulted in the maximum callus biomass. Research has shown that the application of SNPs as fertilizer can improve the growth and quality of plant, such as tomato and *Cinnamomum* *zeylanicum* plants by supporting the metabolic processes involved in cell development [45, 46]. The positive effects of SNPs are likely due to the role of sulfur in forming several biologically active compounds, such as coenzyme A, cysteine, glutathione, and 5-adenylyl sulfate [47, 48]. These compounds help protect cells and can positively impact the quality and viability of callus tissues.

In addition to secondary metabolite elicitors, nanoparticles can also be used as a sources of micronutrients, antimicrobial agents, and stimulators of callus induction [49]. The use of SNPs significantly affected the production of flavonoids, phenolic acid, and ascorbic acid in *L*. *arabicus* callus in the current study; the optimal dose was 100 mg/l. Sulfur is an essential element that has important implications for the biosynthesis and properties of flavonoids and other phenolic compounds containing sulfur in plants [50]. Phenolic acids, flavonoids, and other secondary metabolites play essential roles in regulating growth, morphogenesis, and the production of valuable compounds in plant tissue culture systems [51]. Similarly, various nanoparticle elicitors, such as CuO, and TiO_2_ elicitation, increased the total flavonoid and phenolic contents and improved the bioactive compounds [52]. In agreement with this finding, Tarroum et al. [53] revealed that biogenic zinc oxide nanoparticles enhanced the total phenolic and flavonoid contents in the callus of *Delonix* *elata* and improved bioactive compounds.

To evaluate the metabolic status after SNPs application and secondary metabolites induction, stress markers (MDA and H_2_O_2_) were detected. The findings of this study demonstrated that, in comparison with the control treatment, SNPs concentrations of 25, 50, and 100 mg/l decreased the contents of H_2_O_2_ and MDA, demonstrating the beneficial effects of SNPs in lowering free radicals and enhancing the growth of *L*. *arabicus* L calli. The results provided strong evidence through a negative Pearson correlation between the levels of H_2_O_2_ and MDA, a marker of oxidative stress, and the levels of antioxidant and secondary metabolites in calluses. This decline could result from the green biosynthesis SNPs mediating cell membrane restoration, which enhances callus vigour [54]. However, increasing the concentration of sulfur to 200 mg/l results in the production of a range of free radicals and increases H_2_O_2_ and MDA, which places plants under oxidative stress. Oxidative stress causes by high concentration of metalloids and heavy metals affects several components of cells, most notably the membranes, and oxidizes the lipids in those membranes [55].

Compared with the untreated control, SNPs (100 mg/l) remarkably stimulated PPO. PPO can be involved in the biosynthesis and degradation of metabolites depending on the developmental stage, environmental circumstances and availability and specificity of phenolic compounds [56]. The broadly distributed PPO is known to act on the production of quinones by phenol oxidation where its high activity is connected to plant defense, senescing, wounded, or stressed plant cells [57]. The findings are consisted with the significant Pearson correlation presented in Fig. 8, demonstrating a strong relationship between PPO and the production of secondary metabolites in plants. Previous probes have shown an association between antioxidant activity, flavonoids, and phenolics [58, 59].

Numerous findings indicate a positive correlation between PAL activity and the accumulation of phenolic compounds [60]. In this study, the significant induction in PAL activity was related mainly to the overexpression of the *PAL* gene (Figs. 3, 5). Furthermore, the highest PAL activity and *PAL* gene expression were noted in the callus cultures treated with 100 mg/l SNPs, which may account for the greater accumulation of phenolics and flavonoids in the cultures (Fig. 4). Thus, SNPs may have a positive regulatory effect on activating the phenylpropanoid pathway. The strong Pearson correlation depicted in Fig. 8 provides clear evidence of the observed findings. The enrichment of phenolic chemicals (hydroxybenzoic, hydroxycinnamic, and flavonols) occurred concurrently with this Chung et al. [61]. Similarly, CuO nanoparticles upregulated *PAL* gene expression, confirming the increase in of secondary metabolite production in Chinese cabbage hairy root cultures [61].

The multifold boost metabolite production can be targeted by overexpression of vital genes involved in metabolic pathways. In this study, the expression levels of key genes involved in the biosynthesis of flavonoid (*CHS, CHI* and *FLS*) increased significantly in response to SNPs treatment. The highest expression level of these genes was observed when calli were treated with 100 mg/l SNPs (Fig. 7). High expression levels of genes encoding flavonoid biosynthesis pathway enzymes, such as *CHS, CHI* and *FLS*, have been linked to flavonoid accumulation in callus [62], which is similar to the findings of this study. Similarly, elicitation with CuO NPs was reported to increase the total flavonoid content and upregulate their gene expression (*CHI* and *FLS*) in hairy root cultures of Chinese cabbage. This was consequently accompanied by enrichment of the flavonoid compounds (flavonols, flavonones, and isoflavones) [61]. These findings were supported by HPLC analysis, which revealed that SNPs application significantly increased flavanols (quercetin, rutin, and catechin), flavonones (apigenin and naringenin) and isoflavones (daidzein). Several investigations have indicated that these compounds defend against illnesses caused by excess free radicals, such as cardiovascular diseases, cancer, atherosclerosis, and ischemic disorders [63, 64]. Likewise, magnetite nanoparticles (MNPs) have also been shown to induce the overproduction of catechin, quercetin, and rutin in *Ginkgo biloba* L. callus as a result of the activation of metabolic pathways [64].

Chlorogenic acid (CGA) is regarded as the main bioactive, agent because of its various bioactive functions, including antioxidation, free radical scavenging, antiviral activity, anticancer activity and immunity improvement [65]. CGA refers to a polyphenol ester derivative that is created when quinic acid is combined with hydroxycinnamic acids, such as ferulic acid, caffeic acid, and p-coumaric acid [66]. The biosynthesis of CGA has been proposed to involve the enzyme HQT, which catalyzes the interaction between quinic acid and caffeoyl CoA [67]. Our result revealed that the expression levels of gene involved in chlorogenic acid biosynthetic pathway (*HQT*) significantly changed, especially in the 100 mg/l SNPs (10.11fold); therefore the concentration of chlorogenic acid increased in the 100 mg/l SNPs treatments as measured via HPLC analysis. Similarly, a number of investigations have shown that HQT is a rate-limiting regulatory enzyme in the CGA biosynthetic pathway in various plant species, including artichokes, coffee, and tomatoes [68]. Furthermore, in *L. japonica* calli, there was a positive association between the expression of HQT and the CGA concentration [69]. Additionally, in calli treated with 100 mg/l SNPs, there was a substantial increase in the levels of coumaric acid and caffeic acid compared with those in the control. Caffeic acid is derived from coumaric acid and both have been utilized in the pharmaceutical sector [70]. Therefore, enhanced the production of coumaric acid can potentially be directed towards increased synthesis of caffeic acid and chlorogenic acid [71]. Similarly, ZnO NPs raised the levels of p-coumaric, caffeic, and chlorogenic acids in *Lilium candidum* L. cultures in vitro [72]. In addition, the present data showed highly increased amounts of benzoic acid derivatives, including methyl gallate, gallic acid, and syringic acid under 100 mg/l SNPs treatment, compared with those in the control. These compounds exhibit a variety of biological functions, including anti-inflammatory, antioxidant, and anticancer properties. This finding is in line with Al-Oubaidi and Kasid, [73], who indicated that TiO_2_ nanoparticle caused highly significant production of gallic acid from the callus embryo of *Cicer arietinum* L. Additionally, Chung et al. [61] reported that AgNPs produced a greater amounts of hydroxybenzoic and hydroxycinnamic acids than did the control in bitter gourd cell suspension cultures.

The results of the study revealed a noteworthy increase in the expression levels of the *DXR* gene when *L. arabicus* L was treated with 100 mg/l SNPs. This rise in *DXR* transcript levels likely resulted in an elevation in the functional protein and enzyme activity within *L. arabicus* calli. The findings are consistent with the significant Pearson correlation presented in Fig. 6, demonstrating a strong relationship between the *DXR* expression level and secondary metabolite production in plants. Previous studies have associated *DXR* overexpression with increased terpenoid production, highlighting *DXR* as a key regulator of terpenoid synthesis [74]. In this context, the overexpression of *DXR* has been shown to enhance the accumulation of *Mentha piperita* monoterpenes and essential oils by 50% [16]. The SNPs extract triggered a substantial increase in *DXR* gene expression, indicating that the plant isoprenoid biosynthesis pathway was activated in response to the eliciting treatment.

## Conclusion

The current study presents a groundbreaking application of green biosynthesis sulfur nanoparticles (SNPs) as an ecofriendly callus elicitor. The supplementation of SNPs improved the contents of phenolic, flavonoid, and ascorbic acid in *L. arabicus* L calli, resulting in increasing antioxidant activities. Engrossingly, SNPs enhance the upregulation of eliciting-responsive genes, including *CHS*, *PAL*, *FLS, CHI*, *HQT,* and *DXR*, which are implicated in the synthesis of phenylpropanoids, chlorogenic acid, and terpenoids. Overall, in vitro SNPs supplementation offers a potential elicitor and nutrient source for increasing secondary metabolite and antioxidant activities in* L. arabicus* L callus*.* Consequently, further innovative approaches involving SNPs should be explored for large scale application in many variety of plants tissue culture.

## Data Availability

All data supporting the findings of this study are already presented in this published manuscript and additional supporting files.

## Abbreviations

CHI: Chalcone isomerase
CHS: Chalcone synthase
DXR: Deoxyxylulose phosphate reductoisomerase
FLS: Flavonol synthase
H_2_O_2_: Hydrogen peroxide
HQT: Hydroxycinnamoyl CoA quinate hydroxycinnamoyl transferase *()*
MDA: Malondialdehyde
PAL: Phenylalanine ammonia lyase
PCA: Principal Component Analysis
PPO: Polyphenol oxidase
SNPs: Sulfur nanoparticles
TBA: Thiobarbituric acid
